# Immunomodulatory Activities of *Sambucus javanica* Extracts in DMBA-Exposed BALB/c Mouse

**DOI:** 10.15171/apb.2019.071

**Published:** 2019-10-24

**Authors:** Wira Eka Putra, Muhaimin Rifa'i

**Affiliations:** ^1^Department of Biology, Faculty of Mathematics and Natural Sciences, Universitas Negeri Malang, Indonesia.; ^2^Department of Biology, Faculty of Mathematics and Natural Sciences, Brawijaya University, Indonesia.

**Keywords:** DMBA, BALB/c mice, Immunomodulation, Inflammation, *Sambucus javanica*

## Abstract

***Purpose:*** Accumulating evidence shows the genus of *Sambucus* exerts a broad spectrum of medicinal potencies such as anticancer, antiviral, antibacterial, and antidiabetes. Based on the previous studies, we hypothesized that bioactive compounds of *Sambucus* might alter several biological systems, including the immune system. Therefore, this study extensively aimed to evaluate the immunomodulatory activities of *Sambucus javanica* extracts in 7,12-dimethylbenz[a] anthracene (DMBA)-treated BALB/c mouse.

***Methods:*** The experimental mice were orally administrated with 2.8 mg.kg^-1^ BW of DMBA for ten times within a month. After that, the mice were treated by *S. javanica* berries and leaves extracts for 2 weeks. Subsequently, the inflammation rate was evaluated by using flow cytometry analysis, whereas the necrosis incidences were observed by hematoxylin & eosin staining.

***Results:*** Based on the results, we found the expression of tumor necrosis factor alpha (TNF-α) and interferon gamma (IFN-ɣ) were increased however after treated by *S. javanica* berries and leaves extracts were significantly decreased. In the same way, necrosis incidence was increased in the DMBA-treated group however it was diminished with *S. javanica* extracts treatment.

***Conclusion:*** Together, these results suggested that *S. javanica* extracts have immunomodulatory activities to suppress inflammation and reduce necrosis incidence in experimental mice.

## Introduction

The immune system plays a pivotal role in maintaining the steady state of the biological system. The immune response called immunomodulation has a responsibility to modulate the immune-competent cells against various invaders, malignant cells and any other diseases.^1^ Based on its function, immunomodulation has three types of action namely stimulation, suppression, and restoration of the immune response.^2^ Other studies have been declared that immune-competent cells could be altered by numerous factors such as sex difference, psychological stress, and industrial chemicals pollution.^1,3,4^ Accordingly, a strategy to tackle the undesirable effects from those factors is crucially needed. Nowadays, phytochemicals are recently used as alternative medicine due to their bioactive-rich compounds which potentially modulate the immune system.^5,6^

*Elderberry or Sambucus* is a group of the plant that ubiquitously found in the diverse temperate area. Furthermore, *Sambucus* plant has been known as herbal medicine due to its bioactive compounds’ capabilities to ameliorate multiple types of diseases.^7^ Accumulating evidence determined that *Sambucus* contains several functional bioactive compounds like flavonoids and phenolic acids.^8-10^ Other investigations revealed that *Sambucus* berries and flowers extracts showed the wide spectrum of biological activities such as hematopoiesis modulator, anti-inflammatory, antioxidant, antimicrobial, antiparasitic, antidiabetes and antipyretic effect.^6,11-13^

Recently, the investigation of the effects of natural compounds toward the cellular and molecular mechanism involved in the biological system including disease is a precedence for the present-day research. In advance, the evidence about the immunomodulation effects of *Sambucus javanica* is based on very limit data. Therefore, in this present study, we aim to emphasize the finding of the modulatory effects of *S. javanica* extracts toward the immune system.

## Materials and Methods

### Materials preparation

Plant materials such as berries and leaves of *S. javanica* were obtained from Materia Medica Batu, the Ministry of Health Indonesia. Then, to obtain the crude extract, both berries and leaves were proceeded by using ethanol extraction. Firstly, the berries and leaves simplicia were soaked in 70% ethanol for 5 days. The step then continued by macerate filtration. After that, the total yield was evaporated to elucidate the ethanol as the solvent. Finally, the extracts were carried out at 4°C room storage for further experiment.

### Animal care and treatment groups

In this present study, pathogen-free BALB/c mice were purchased from Gadjah Mada University Animal Laboratory. The three-month-old female mice were acclimated for 1 week before experimental treatment. Experimental group were divided into 8 clusters that consist of vehicle group; DMBA group, DMBA 2.8 mg.kg^-1^ BW; BD1 group, DMBA 2.8 mg.kg^-1^+ berries extracts 200 mg.kg^-1^ BW; BD2 group, DMBA 2.8 mg.kg^-1^ + berries extracts 400 mg.kg^-1^ BW; BD3 group, DMBA 2.8 mg.kg^-1^ + berries extracts 800 mg.kg^-1^ BW; LD1 group, DMBA 2.8 mg.kg^-1^ + leaves extracts 200 mg.kg^-1^ BW; LD2 group, DMBA 2.8 mg.kg^-1^ + leaves extracts 400 mg.kg^-1^ BW; LD3 group, DMBA 2.8 mg.kg^-1^+ leaves extracts 800 mg.kg^-1^ BW. The treatment procedures started from DMBA (Tokyo Chemical Industry Co. Ltd.) administration to the experimental animal by dose of 2.8 mg.kg^-1^ BW for 10 times administration in a month then followed by *Sambucus* extracts treatment every day for two weeks with the various doses such as 200, 400, and 800 mg.kg^-1^ BW.

### Immunostaining and flow cytometry

Flow cytometry analysis was performed to evaluate inflammation rate in an experimental animal through measuring the relative number of tumor necrosis factor alpha (TNF-α) and interferon gamma (IFN-ɣ). Specifically, isolated spleen was homogenized and was stained using several antibodies such as anti-CD4, anti-TNF-α, and anti-IFN-ɣ antibody (Biolegend). Together with that, FACS Calibur^TM^ were employed to evaluate the expression of CD4^+^ TNF-α^+^ and CD4^+^IFN-ɣ^+^.^14,15^

### Tissue preparation and hematoxylin & eosin staining

As similar as the previous study, the staining procedures started by specimen deparaffinization with xylol and ethanol respectively. After that, the tissue section of liver and renal cortex were stained with hematoxylin and eosin for 3 minutes. Stained samples then cleaned and dehydrated by using tap water and xylol respectively. Finally, each sample was mounted with malinol and was covered by cover glass.^16^

### Statistical analysis

The student *t* test was used to review and compare the statistical difference between two different groups. Thus, in this study, a value of *P* <0.05 was considered significant between two different groups. The data were expressed as means ± standard deviation.

## Results and Discussion

**Figure 1:**
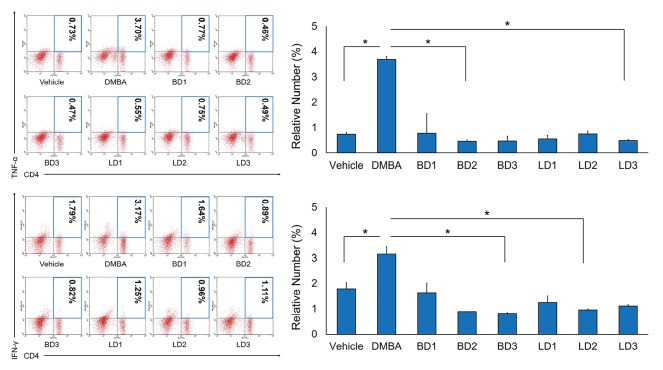
*Sambucusjavanica* extracts reduced the relative number of CD4^+^TNF-α^+^cells and CD4^+^IFN-ɣ^+^cells. The bars are calculation of the relative number of CD4^+^TNF-α^+^cells and CD4^+^IFN-ɣ^+^cells expression respectively. The results were represented as the mean ± SD. *P <0.05, indicate significant difference. Treatment group in this study were vehicle; DMBA 2.8 mg.kg^-1^ BW; BD1, DMBA 2.8 mg.kg^-1^+ berries extracts 200 mg.kg^-1^ BW; BD2, DMBA 2.8 mg.kg^-1^ + berries extracts 400 mg.kg^-1^ BW; BD3, DMBA 2.8 mg.kg^-1^ + berries extracts 800 mg.kg^-1^ BW; LD1, DMBA 2.8 mg.kg^-1^ + leaves extracts 200 mg.kg^-1^ BW; LD2, DMBA 2.8 mg.kg^-1^ + leaves extracts 400 mg.kg^-1^ BW; LD3, DMBA 2.8 mg.kg^-1^+ leaves extracts 800 mg.kg^-1^ BW.

Inflammation rate which evaluated by measuring the relative number of TNF-α and IFN-ɣ was analyzed by flow cytometry after treatment of DMBA or *S. javanica* berries and leave extracts in experimental mice. According to the analyzed results, the relative number of TNF-α and IFN-ɣ which produced by CD4 helper T-cells was increased by DMBA treatment. Otherwise, the treatment of *S. javanica* berries and leave extracts significantly decreased the inflammation rate (Figure 1).

**Figure 2:**
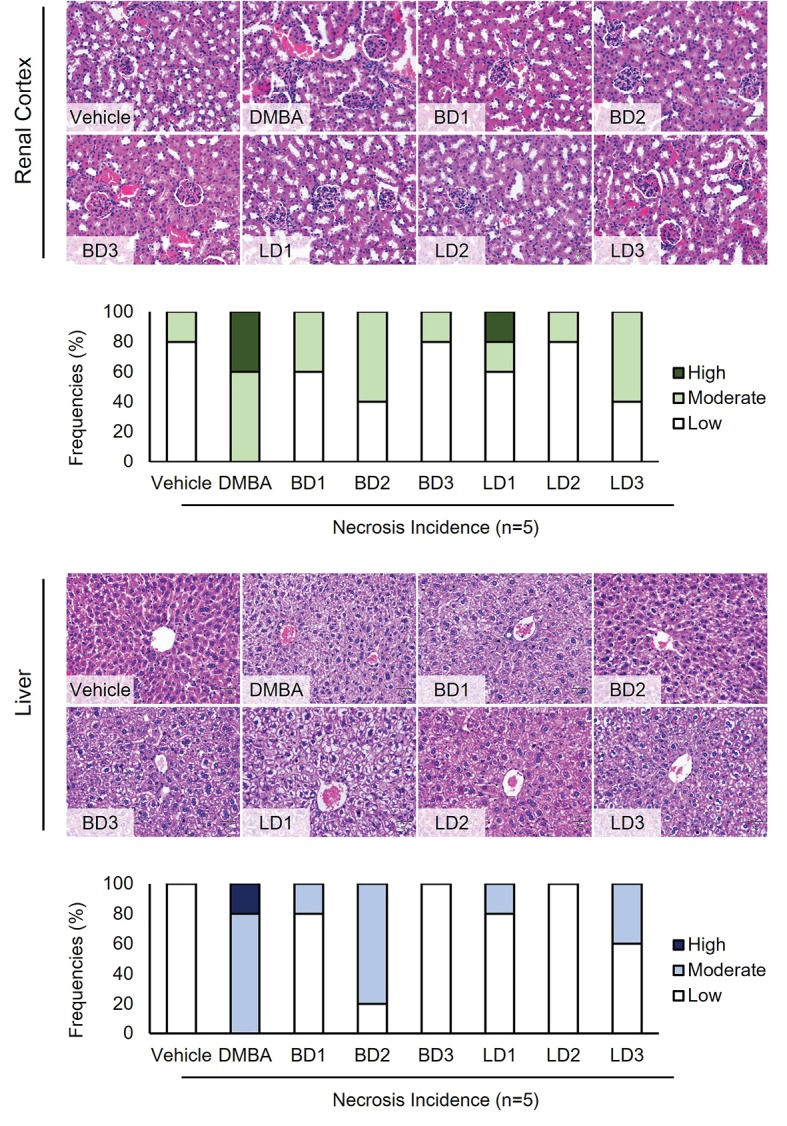
Representative microphotograph of necrosis incidence in renal cortex and liver tissues from experimental mice (HE Staining, M=400×). The DMBA-treated mice showed more numbers of necrosis incidence comparing to the *S. javanica* extracts treatment. Moreover, the treatment group in this study were vehicle; DMBA 2.8 mg.kg^-1^ BW; BD1, DMBA 2.8 mg.kg^-1^+ berries extracts 200 mg.kg^-1^ BW; BD2, DMBA 2.8 mg.kg^-1^ + berries extracts 400 mg.kg^-1^ BW; BD3, DMBA 2.8 mg.kg^-1^ + berries extracts 800 mg.kg^-1^ BW; LD1, DMBA 2.8 mg.kg^-1^ + leaves extracts 200 mg.kg^-1^ BW; LD2, DMBA 2.8 mg.kg^-1^ + leaves extracts 400 mg.kg^-1^ BW; LD3, DMBA 2.8 mg.kg^-1^+ leaves extracts 800 mg.kg^-1^ BW. Semi-quantitative measurement was applied to cluster the samples as high, moderate, or low of necrotic cells incidence. The bars are the frequencies of necrotic cells incidence in lung samples of mice lung cancer model.

The histological analysis performed to assess the effect of DMBA and bioactive compounds of *S. javanica* after induction in an experimental animal and to clarify the necrosis incidence in each group of treatment. In this present study, we applied semi-quantitative analysis which group the necrosis incidence into three parts, i.e. high, medium, and low incidence. Based on the experiment, DMBA promoted necrosis incidence in the liver, however it reduced after the animal model treated by *S. javanica* extracts. Similar to that, necrosis incidences in the renal cortex were promoted by DMBA induction compared to *S. javanica* berries or leave extracts (Figure 2).

**Figure 3:**
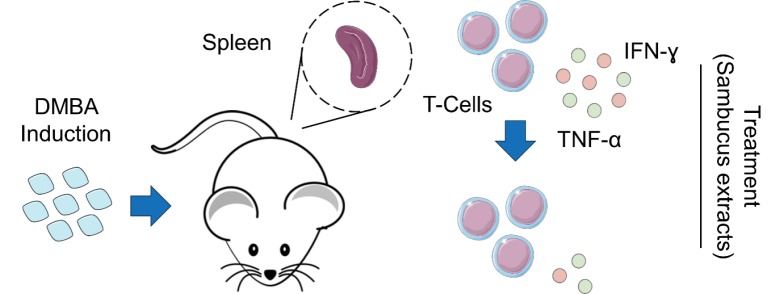
A schematic diagram showed DMBA administration in BALB/c mouse induces inflammation, however immunomodulation activity of *S. javanica* suppressed it by reducing pro-inflammatory cytokines production.

DMBA is a member of polycyclic aromatic hydrocarbons that abundantly found in the environment as a part of pollutant. DMBA has multiple adverse effects and act as a toxic and carcinogenic agent by inducing the production of reactive oxygen species (ROS) resulting in the increasing level of lipid peroxidation, promoting DNA damage, and depleting the antioxidant system of the cell.^17,18^ Currently, we observed the immunomodulation effects of *S. javanica* extracts toward DMBA-administrated mouse by evaluating the inflammation rate and necrosis incidence (Figure 3).

In this present study, we revealed that *S. javanica* extracts can reduce the relative number of TNF-α. Consistent with that, IFN-ɣ also suppressed after the treatment of the extract in an experimental model (Figure 1). Together, these results suggest that bioactive compounds of *S. javanica* might reduce inflammation status in DMBA-administrated mouse. Identically, several reports also showed the anti-inflammatory effects of phenolic acids and flavonoid from medicinal plants crude extracts such as *Gymnema sylvestre* and *Alternanthera tenella*.^19,20^ Other investigations about *Sambucus* plant, commonly *S. nigra* and *S. ebulus*, determined that their bioactive compounds have been effectively used as antioxidant, antiviral, and antidiabetes.^12,13,21^ Molecular mechanism showed that polyphenols such as luteolin and quercetin suppressed lipopolysaccharide expression which in turn promoted the activation of mitogen-activated protein kinases signalling pathway including p38 and extracellular signal-regulated kinases pathways, therefore, reducing the production of proinflammatory cytokines and leukocyte.^5,22^

Both liver and renal are the most important excretory organs which play a crucial role in detoxification of some toxic metabolic waste products. DMBA induced hepatorenal dysfunction in rats. According to Dakrory et al and Sharma et al, several hallmarks for nephrotoxicity such as plasma level of urea, creatinine, and uric acid were increased in DMBA-treated rats.^23,24^ Similarly, in the liver case, DMBA showed the increasing number of liver malondialdehyde (MDA) and the decreasing number of glutathione, glutathione-S-transferase, superoxide dismutase, and catalase which all of those compounds would worsen the condition of the liver.^23-25^

According to our observation, we found that DMBA treatment enhances the necrosis incidence both in the liver and renal cortex tissues. Whereas, the *S. javanica* extracts dramatically decreased the number of necrosis incidence (Figure 2). According to these results, we implied that bioactive compounds bearing in *S. javanica* exert their effects to reduce necrosis incidence in experimental mice. Comparatively, an investigation reported that *Punica granatum* which contains rich antioxidants including tannins and flavonoids had been known to decrease oxidative stress in DMBA-administrated rats.^26^ Another study related to the medicinal plant also showed that *Moringa oleifera* crude extract has a protective effect in the liver and renal tissue against DMBA toxicity.^24^

## Conclusion

Based on the above results, it suggests that *S. javanica* extracts decreased the inflammation rate in experimental mice. Moreover, the extracts also induced the decline of necrosis incidence in both liver and renal tissues in DMBA-administrated BALB/c mice. Finally, emphasizing the observations about the medicinal properties of *S. javanica* is still needed for a future therapeutic strategy against other diseases.

## Ethical Issues

All experiment aspects of this study have been evaluated by Brawijaya University, Research Ethics Committee with legal number 312-KEP-UB.

## Conflict of Interest

We declare there is no conflict of interest in this study.

## Acknowledgments

Authors thank the members of Laboratory of Animal Anatomy and Physiology, Brawijaya University for help in this experiment. WEP thanks to LPDP (Indonesia Endowment Fund for Education) for support this study.
